# Potential of Epicardial Adipose Tissue Quantification in Predicting Atrial Thrombus in Patients With Atrial Fibrillation

**DOI:** 10.31083/RCM37467

**Published:** 2025-10-28

**Authors:** Huan Wang, Tianze Wang, Manyun Tang, Peizhu Dang, Changying Zhao, Yang Yan, Jianying Li, Tao Shi, Jianxin Guo

**Affiliations:** ^1^Department of Radiology, The First Affiliated Hospital of Xi’an Jiaotong University, 710061 Xi’an, Shaanxi, China; ^2^Department of Neurosurgery, The First Affiliated Hospital of Xi’an Jiaotong University, 710061 Xi’an, Shaanxi, China; ^3^Department of Hepatobiliary Surgery, The First Affiliated Hospital of Xi’an Jiaotong University, 710061 Xi’an, Shaanxi, China; ^4^Department of Cardiovascular Medicine, The First Affiliated Hospital of Xi’an Jiaotong University, 710061 Xi’an, Shaanxi, China; ^5^Department of Cardiovascular Surgery, The First Affiliated Hospital of Xi’an Jiaotong University, 710061 Xi’an, Shaanxi, China; ^6^GE Healthcare, Computed Tomography Research Center, 100000 Beijing, China

**Keywords:** epicardial adipose tissue, prediction, intra-atrial thrombus, atrial fibrillation

## Abstract

**Background:**

Atrial fibrillation (AF) can increase the risk of stroke by five-fold; strokes associated with AF are more likely to lead to death or severe disability in patients. Thus, preventing the formation of thrombosis is of vital importance in the treatment of patients with AF. Epicardial adipose tissue (EAT) is a risk factor for AF and is closely associated with many AF-related complications. However, to our knowledge, no in-depth studies on the relationship between the incidence of thrombosis in AF patients and EAT have been conducted. Therefore, it is of great clinical significance to explore the potential of EAT quantification in predicting intra-atrial thrombosis in patients with AF.

**Methods:**

This is a case–control study; patients with AF who underwent coronary computed tomography angiography (CCTA) were included. These patients were divided into the thrombus group and the non-thrombus group according to the results of transesophageal echocardiography (TEE). The volume of EAT, the mean density of EAT, and the ratio of EAT volume to the whole heart volume were measured by CCTA, and the data of the two groups were compared. Meanwhile, the diagnostic efficiency of using these parameters was analyzed.

**Result:**

A total of 308 patients with AF who underwent both TEE and CCTA were enrolled in this study. After a 1:1 propensity score matching (PSM) analysis based on age and sex, a total of 76 patients were finally included. Compared with the patients in the non-thrombus group, those in the thrombus group had a larger volume of EAT (132.38 ± 45.25 cm^3^ vs. 95.51 ± 25.38 cm^3^; *p* < 0.001) and a higher ratio of EAT volume to the whole heart volume (0.13 ± 0.05 vs. 0.10 ± 0.03; *p* < 0.05). However, there was no difference in the mean density of EAT between the two groups. The volume of EAT was identified as an independent risk factor (odds ratio = 1.042; *p* = 0.003). Moreover, the receiver operating characteristic (ROC) analysis presented the EAT volume as a potential diagnostic value in predicting intra-atrial thrombus in AF patients, with an area under the curve (AUC) of 0.755.

**Conclusions:**

The EAT volume may be a potential biomarker for predicting intra-atrial thrombosis in patients with AF; however, further validation is required to confirm the diagnostic value.

## 1. Introduction

Atrial fibrillation (AF) is a cardiovascular disease associated with a higher risk of complications and mortality [1]. Epidemiological evidence has shown that the incidence and prevalence of AF have increased significantly in the past 20 years [2, 3]; one of the most concerning complications associated with AF is a stroke caused by left atrial thrombus [4]. Cardiomyopathy often accompanies the later stage of AF. Through various mechanisms such as inflammation, oxidative stress, and stretching, it will further lead to fibrosis, electrophysiological and autonomic nerve remodeling, as well as a pre-thrombotic state. The complex interactions among these mechanisms increase the risks of stroke and other thromboembolic events. Previous studies have shown that AF can increase the risk of stroke by five-fold [5, 6], and the strokes associated with AF are more likely to be fatal or severely incapacitating [2, 3]. AF is estimated to be responsible for 15% of all strokes worldwide. Thus, a more comprehensive understanding of this association and the development of intensive stroke prevention measures are needed. Moreover, preventing thrombosis in the treatment of patients with AF is critical.

Obesity is an independent risk factor for AF [7]. An increasing amount of evidence indicates that epicardial adipose tissue (EAT), as a manifestation of visceral obesity, is considered to be closely associated with the occurrence and development of AF [8, 9, 10]. EAT is a type of visceral adipose tissue deposited on the surface of the heart, which has special anatomy, physiology, biochemistry, and the characteristics of secreting various adipose cytokines [11, 12]. Furthermore, EAT can affect the myocardium and coronary artery through paracrine regulation and the capillary mechanism, thus affecting cardiac and metabolic functions. Many studies have concluded that EAT is a risk factor for AF and is closely related to many AF complications [13, 14]. However, to our knowledge, no in-depth study on the relationship between the incidence of thrombus formation in AF patients and EAT has previously been performed.

Although transesophageal echocardiography (TEE) represents the gold standard for thrombosis after AF, TEE is an invasive examination and is not suitable for large-scale implementation. Therefore, coronary computed tomography angiography (CCTA) is a commonly used clinical cardiac examination method with the characteristics of being non-invasive, safe, and cheap. Consequently, using EAT in CCTA images is of great significance to quantitatively predict atrial thrombus in patients with AF.

This study recruited AF patients who had received both CCTA and TEE and compared EAT characteristics among patients with and without intra-atrial thrombus to investigate the potential of EAT quantification in predicting intra-atrial thrombus in AF patients.

## 2. Methods

### 2.1 Study Population

**Fig. 1.**
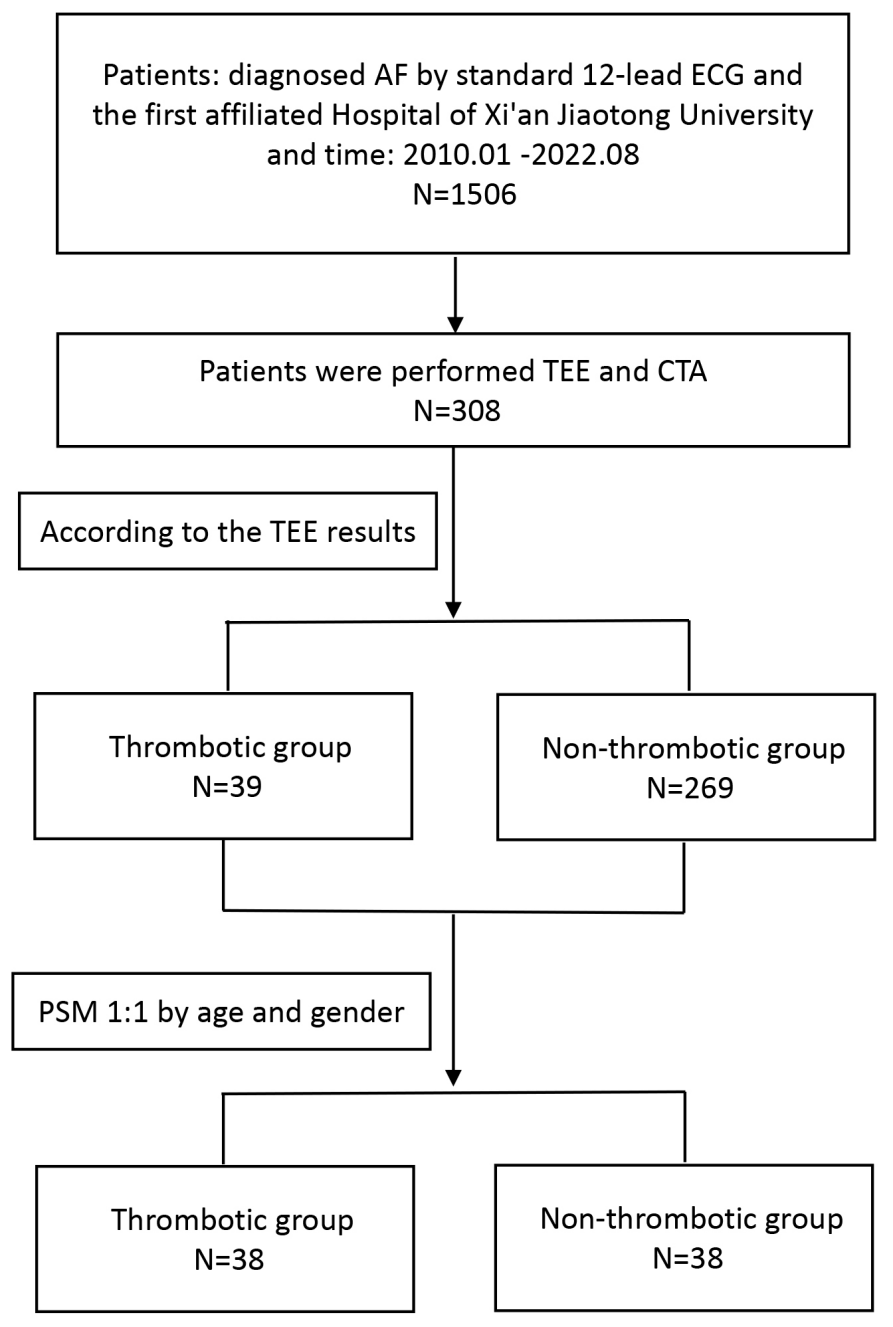
**Flowchart of the EAT volume for predicting thrombotic events in AF patients**. Note: AF, atrial fibrillation; TEE, transesophageal echocardiography; CTA, computerized tomography angiography; PSM, propensity score matching; EAT, epicardial adipose tissue; ECG, electrocardiogram.

This was a single-center, retrospective, case–control study that enrolled patients with AF who received TEE and CCTA examinations in our hospital between January 2010 and August 2022. The inclusion criteria included the following: (1) patients were clinically diagnosed with AF by standard 12-lead electrocardiogram (ECG) or 24 h Holter monitor, (2) patients underwent TEE and CCTA examinations within one week. The exclusion criteria included patients with incomplete clinical and imaging information. The subjects were then divided into the thrombus group and the non-thrombus group according to the TEE results. To control for bias, the final number of patients included in each group was determined after a 1:1 propensity score matching (PSM) analysis based on age and sex (Fig. 1).

This study was approved by the Ethics Committee of our hospital (NO. XJTU1AF2023LSK-168). Since this study was observational in nature, the requirement for patient-informed consent was waived.

### 2.2 Computed Tomography Scanning Scheme and Image Analysis

**Fig. 2.**
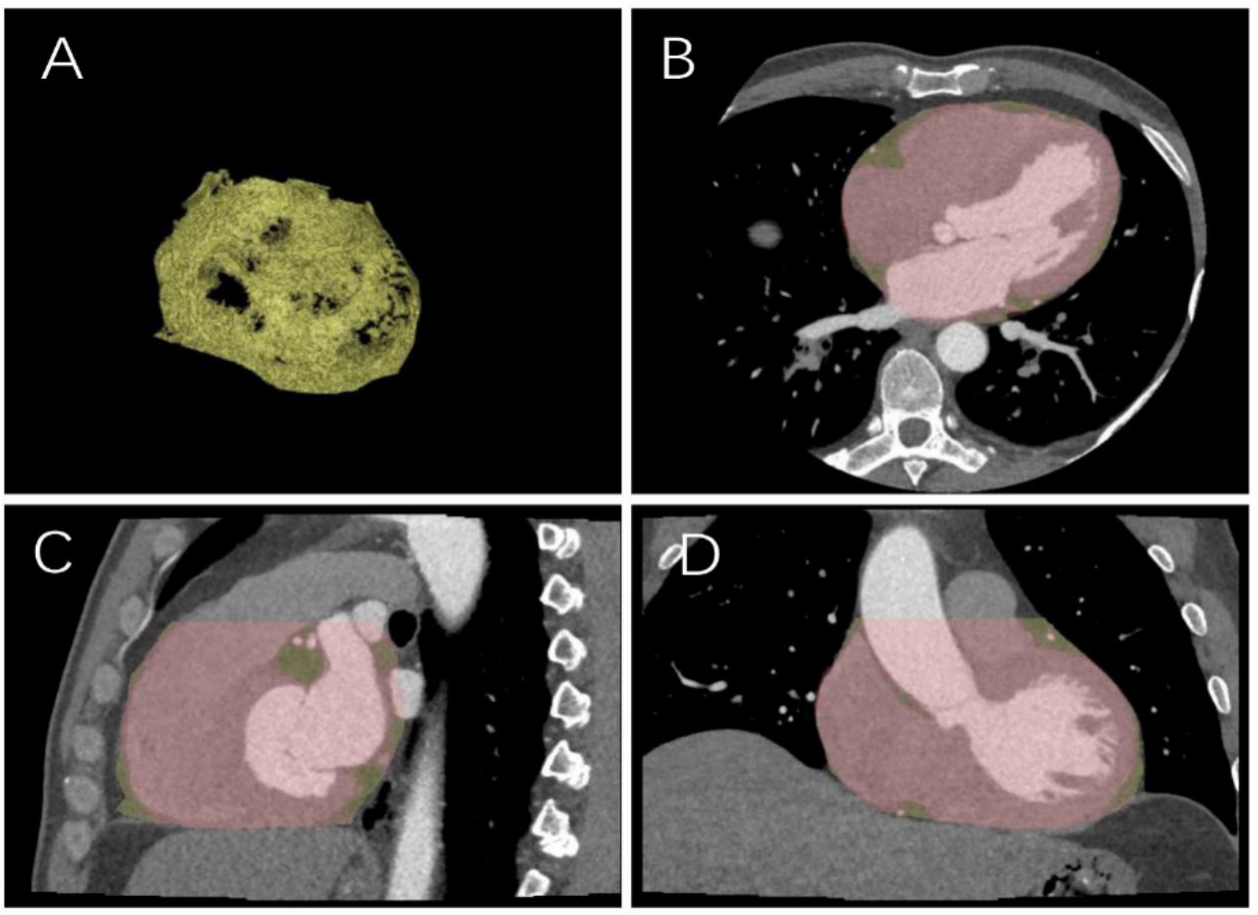
**Sketch diagram of epicardial fat**. (A) Epicardial fat diagram. (B) Axial cardiac image. (C) Sagittal cardiac image. (D) Coronal cardiac image.

All CCTA scans were conducted using a 256-slice computed tomography (CT) scanner with a 160 mm-wide detector collimation (Revolution CT, GE HealthCare, Waukesha, WI, USA). The scans were performed in a prospective electrocardiogram-triggered cardiac scan mode and completed within one heartbeat. All subjects were scanned in a free-breathing state. Patients were required to remain stable and take shallow breaths during the scanning process. The scanning parameters were as follows: tube voltage: 120 kV; tube current: smart mA (100–700 mA); gantry rotation speed: 0.28 s per revolution; detector width: adjusted between 120 and 160 mm according to the heart size of the patient. A total of 40–50 mL of the non-ionic contrast agent Iopamidol (350 mgI/mL) was injected into the anterior cubital vein with a high-pressure syringe at a flow rate of 4–5 mL/s, followed by the injection of 30 mL of 0.9% sodium chloride solution at the same flow rate. The bolus tracking technique was employed, and a region of interest (ROI) was selected at the aortic level to monitor the increase in the CT value. The scan was triggered when the CT value in the ROI exceeded 220 Hounsfield units (HUs), and the actual scan started after a delay of 1.6 s. A dedicated workstation (Advanced Workstation 4.6, GE HealthCare) was used to reconstruct the CCTA images with an image slice thickness of 0.625 mm. Two experienced radiologists (both with over 20 years of experience in reading CCTA images) separately reconstructed the appropriate cardiac phase images in three dimensions on a post-processing workstation (Advanced Workstation 4.6, GE HealthCare, Beijing, China), and manually outlined the epicardial boundary layer by layer using the VolumeViewer software (Shanghai United Imaging Healthcare, Shanghai, China). The two radiologists conducted the relevant measurements separately. The specific steps were as follows: (1) The approximate range of adipose tissue around the heart was delimited with a rectangular frame. The upper margin was at the level of the pulmonary valve, the lower margin was at the level of the diaphragm, the front reached the sternum, and the back reached the esophagus; (2) a manual depiction of the boundary along the pericardium every five layers in the cross-section; (3) defined CT value range of the EAT as –190 to –30 HUs; (4) the VolumeViewer software generated the EAT volume, the mean density of the EAT, and the whole heart volume, and the EAT volume/whole heart volume ratio was calculated (Fig. 2). If the measurement results of the two radiologists showed good consistency (with the intra-class correlation coefficient (ICC) >0.75, referring to the section of statistical analysis), the average value was used; otherwise, a third radiologist would make the measurements again, and the average value of the measurements of the three radiologists would be used as the final result.

### 2.3 Statistical Analysis 

Statistical analysis was performed using SPSS software (version 26, IBM, Armonk, NY, USA). PSM was conducted based on gender and age to reduce bias, with study subjects matched at a 1:1 ratio. If normality was satisfied for quantitative data, the data were presented as the mean (± standard deviation). Data with non-normal distribution were described using the median and interquartile range (Q25, Q75). Independent sample tests—the *t*-test and Mann–Whitney U test—were used to compare the two groups. Qualitative data were expressed as numbers (percentages), and the chi-square test was applied for between-group comparisons. The ICC was used to test the consistency of the measurement results between the two radiologists. An ICC value greater than 0.75 indicated good reliability and consistency between the two sets of data. Conditional logistic regression was used to analyze the influencing factors of thrombus formation in AF. Receiver operating characteristic (ROC) curves were used to analyze the ability of various factors to predict thrombus formation in patients with AF. All tests were two-tailed, and a *p*-value < 0.05 was considered statistically significant.

## 3. Results

### 3.1 Baseline Characteristics

A total of 308 patients were initially considered. After PSM, 76 patients were finally enrolled, 38 of whom were in the thrombus group; the baseline characteristics are shown in Table 1. There was no significant difference in age, sex, body mass index (BMI), diabetes mellitus (DM), hypertension (HTN), coronary heart disease (CHD), or oral anticoagulant (OAC) use. In the thrombus group, the EAT volume (132.38 ± 45.25 cm^3^ vs. 95.51 ± 25.38 cm^3^; *p* < 0.001) and the ratio of EAT volume to whole heart volume (0.13 ± 0.05 vs. 0.10 ± 0.03; *p* = 0.004 < 0.05) were significantly higher than those in the non-thrombus group; however, there was no difference in the mean density of the EAT between the two groups (–82.56 ± 7.01 HUs vs. –80.89 ± 5.97 HUs; *p* = 0.224 > 0.05).

**Table 1.** **The comparison of baseline characteristics between the thrombus group and the non-thrombus group among atrial fibrillation patients**.

Variable	Atrial fibrillation with thrombus group	Atrial fibrillation with non-thrombus group	*p*-value
Sex (male/female)	38 (20/18)	38 (23/15)	0.494
Age (y)	64.92 ± 7.93	65.29 ± 7.21	0.833
BMI (kg/m^2^)	24.33 ± 3.05	25.54 ± 3.48	0.113
EAT (cm^3^)	132.38 ± 45.25	95.51 ± 25.38	<0.001
Ratio	0.13 ± 0.05	0.10 ± 0.03	0.004
HU	–82.56 ± 7.01	–80.89 ± 5.97	0.224
DM	0.89 ± 0.31	0.95 ± 0.23	0.402
HTN	0.71 ± 0.46	0.50 ± 0.51	0.062
CHD	0.95 ± 0.23	0.89 ± 0.31	0.042
OACs	0.68 ± 0.47	0.42 ± 0.50	0.021

Ratio, EAT volume-to-whole heart volume ratio; HU, Hounsfield unit; BMI, body mass index; DM, diabetes mellitus; HTN, hypertension; CHD, coronary heart disease; OACs, oral anticoagulants; EAT, epicardial adipose tissue.

### 3.2 Consistency Analysis

The ICC test was used for the intra-group correlation to examine the consistency of the data measured by the two radiologists; the ICC value was 0.980 (Table 2).

**Table 2.** **Comparison of inter-rater agreement**.

	Intraclass correlation	95% confidence interval	
		Lower bound	Upper bound
Single measure	0.961	0.936	0.977
Average measure	0.980	0.967	0.988

### 3.3 Logistic Regression Analysis

When the occurrence of thrombosis was used as the dependent variable, and gender, age, BMI, DM, HTN, CHD, OAC, EAT volume, average density, and the ratio of EAT volume to whole heart volume were applied as independent variables, logistic regression analysis identified that the EAT volume was an independent risk factor for the formation of intra-atrial thrombus in patients with AF (Table 3).

**Table 3.** **Result of logistic regression analysis**.

Variables in the Equation							
	B	S.E.	Wald	Sig	Exp(B)	95% Confidence Interval	
						Lower	Upper
Sex (1)	0.574	0.594	0.932	0.334	1.775	0.554	5.690
Age	–0.037	0.037	0.986	0.321	0.964	0.896	5.690
EAT	0.041	0.014	8.898	0.003	1.042	1.014	1.037
Ratio	–5.397	12.451	0.188	0.665	0.005	0	1.037
HU	0.070	0.059	1.408	0.235	1.073	0.955	1.071

Variable(s) entered on step 1: sex (1: female, 2: male), age (y), EAT (EAT volume in cm^3^), ratio (EAT volume-to-whole heart volume ratio), HU (average EAT density). EAT, epicardial adipose tissue; HU, Hounsfield unit.

### 3.4 ROC Curve Analysis

**Fig. 3.**
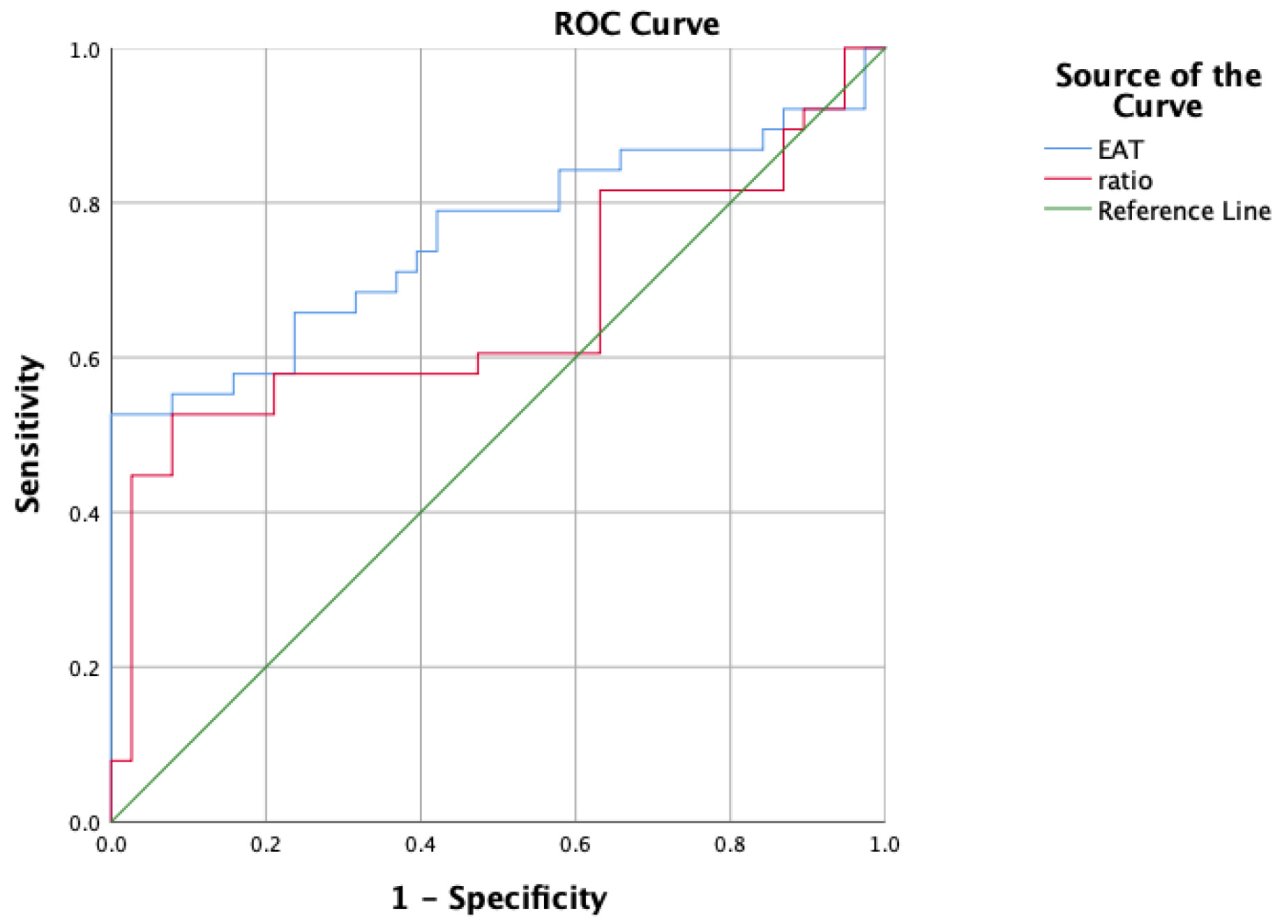
**ROC curve for predicting thrombotic events in AF patients using EAT volume**. Note: ratio, EAT volume-to-whole heart volume ratio; ROC, receiver operating characteristic; AF, atrial fibrillation; EAT, epicardial adipose tissue.

By analyzing the ROC curves and comparing the area under the curve (AUC) (Fig. 3 and Table 4), the EAT volume (AUC = 0.755) and the ratio of EAT volume to whole heart volume (AUC = 0.661) demonstrated potential diagnostic values in predicting intra-atrial thrombus in patients with AF. Upon calculation, the sensitivity and specificity of EAT volume in predicting thrombus formation after AF at a value of 137.05 cm^3^ were 52.6% and 99%, respectively. This indicates that compared with other patients, those with an EAT volume ≥137.05 cm^3^ have a significantly higher possibility of thrombus formation after AF.

**Table 4.** **Predictive efficacy analysis of the area under the curve**.

Test result variable(s)	Area	95% confidence interval	
		Lower bound	Upper bound
EAT	0.755	0.641	0.868
Ratio	0.661	0.532	0.789

EAT, epicardial adipose tissue.

## 4. Discussion

This study explored the potential of quantifying EAT in predicting intra-atrial thrombus in patients with AF through logistic regression analysis and ROC curves. Our findings indicate that the EAT volume is an independent risk factor and exhibits good diagnostic efficacy in predicting thrombus formation in AF patients. Additionally, we discovered that the ratio of EAT volume to the whole heart volume is higher in AF patients with thrombus. However, the logistic regression analysis reveals that this ratio is not an independent risk factor. Many studies [15, 16] have highlighted that the EAT is a risk factor for AF. Zhu *et al*. [10] conducted a systematic review and analysis of the relationship between the EAT volume and AF. The study [17] showed that in ten case studies, both the total EAT volume and the EAT volume around the left atrium in patients with AF increased significantly; the amount of EAT may be associated with an increased risk of AF. Kogo *et al*. [11] surveyed 77 patients who underwent cardiac surgery and found that there was a strong association between high EAT volume and the occurrence of postoperative AF (POAF), confirming the relationship between EAT and AF. However, EAT volume as a risk factor for thrombosis after AF remains under debate. A study [18] has found that EAT may be involved in the formation of thrombus in the pre-thrombus state in patients with AF. Liu *et al*. [12] conducted a study on 90 patients with non-valvular AF who had undergone radiofrequency catheter ablation. Baseline data and concomitant diseases were recorded, and indicators of the coagulation status were measured. Meanwhile, the total EAT volume (EAT-total) and the EAT volume around the left atrium (EAT-LA) were simultaneously recorded. A statistical analysis was conducted on the correlations between EAT-total, EAT-LA, and the blood coagulation status indicators. The analysis presented that both EAT-total and EAT-LA were positively correlated with D-dimer and fibrinogen (FIB), and there was an independent association. The study [19] suggests that EAT in patients with AF may be involved in the formation of the pre-thrombotic state during AF, and the EAT volume may be an independent risk predictor for thromboembolism in patients with AF. Ju *et al*. [13] studied the factors of thrombosis recurrence in patients after surgical treatment and highlighted that EAT was significantly higher after one-stop treatment, suggesting that EAT may be a risk factor for increased thromboembolic events after one-stop treatment. The Ju *et al*. [13] study shares certain similarities with ours, which provides a starting point and theoretical support for our research. We studied the occurrence of untreated thromboembolism in our patients, with a greater emphasis on prevention than in previous studies, hoping to demonstrate that EAT prevents a factor in thrombosis.

At present, the mechanism through which EAT leads to thrombosis in AF patients remains unclear. Nonetheless, there is growing evidence that inflammation may be the cause of how EAT affects thrombosis after AF. Indeed, prior studies [20, 21] have found that EAT has strong biological activity and can secrete various proinflammatory factors. The inflammatory response and activation of coagulation do not work independently, but cooperate in a complex and synchronous manner [22]; meanwhile, inflammation has been shown to cause endothelial dysfunction, promote the formation of a pro-thrombus state, and lead to the occurrence of thromboembolism [9]. Mazurek *et al*. [9] observed EAT glucose metabolism using electron emission computed tomography and found that the EAT glucose metabolism of patients in the AF group was significantly higher than that in the control group, which confirmed that the EAT in patients with AF had higher inflammatory activity compared with normal people. In addition, some scholars [23] have reported that EAT thickness in patients with AF is significantly correlated with the ratio between neutrophils and lymphocytes, which is a reliable indicator of inflammation. Therefore, there is reason to suspect that EAT in patients with non-valvular AF is involved in the formation of a pre-thrombus state in AF and promotes the formation of thrombus in AF by causing local and systemic inflammation.

In addition, we speculate that the impact of EAT on thrombus formation in patients with AF may be related to the duration of AF. Nagashima *et al*. [24] found that patients with persistent AF had a larger volume of EAT and higher levels of high-sensitivity C-reactive protein and interleukin-6 compared to patients with paroxysmal AF. Some studies [25, 26, 27, 28] have also shown that patients with persistent or permanent AF have a higher risk of stroke than those with paroxysmal AF, and the duration of AF may influence thrombus formation. Therefore, we believe that the higher incidence of thrombus formation in patients with persistent AF may be due to the significant increase in the level of C-reactive protein, which is an important indicator of inflammation. We hope to investigate further whether thrombus formation in patients with AF is related to the duration of AF and the level of inflammation in the future. We acknowledge that there are some limitations in our study. Firstly, this was a single-center, retrospective, observational study, with a small sample size in the thrombus group. Secondly, we performed the manual delineation of EAT in the workstation, which could have introduced a slight error. Although we tested the consistency, we still actively explored the realization of artificial intelligence automatic delineation to reduce the bias further. Finally, EAT alone cannot completely exclude or confirm the existence of intra-atrial thrombus. To provide more accurate predictions, increased data and other clinical markers are needed to enhance and refine the prediction in the future.

## 5. Conclusions

The EAT volume may serve as an important imaging marker for predicting intra-atrial thrombosis in patients with AF. This finding provides a new perspective for assessing the risk of thrombosis in AF patients and is expected to assist clinicians in accurately identifying those at high risk of intra-atrial thrombosis, thus enabling early warning and the formulation of personalized thrombosis prevention and treatment strategies. In the future, it is necessary to incorporate more evaluation parameters and expand the sample size to conduct in-depth research, to provide more solid evidence to support the precision medicine of AF patients with thrombosis-related complications.

## Availability of Data and Materials

All data generated or analysed during this study are included in this published article.
